# Novel Bacterial Biocontrol Agents for Sustainable Management of Olive Mite Pests in Saudi Arabia

**DOI:** 10.3390/plants15091307

**Published:** 2026-04-24

**Authors:** Mahmoud M. Al-Azzazy, Saleh S. Alhewairini, Medhat Rehan

**Affiliations:** 1 Department of Plant Protection, College of Agriculture and Food, Qassim University, Buraydah 51452, Saudi Arabia; hoierieny@qu.edu.sa; 2 Department of Plant Production, College of Agriculture and Food, Qassim University, Buraydah 51452, Saudi Arabia

**Keywords:** biological control, biopesticide, *Bacillus subtilis*, *Paenibacillus tundrae*, *Streptomyces tricolor*, sustainability

## Abstract

The olive tree (*Olea europaea* L.) is one of the oldest known cultivated trees worldwide and an iconic species within the Mediterranean Basin. This study evaluated the impact of three bacterial strains, *Bacillus subtilis* D3, *Paenibacillus tundrae* M4, and *Streptomyces tricolor* HM10, on the mortality of the following four mite pests: *Oxycenus niloticus*, *Tegolophus hassani*, *Aceria olivi*, and *Tetranychus urticae*. *B. subtilis* D3 confirmed the highest efficacy, causing 91.84–85.36% mortality in laboratory tests and 88.90–84.12% in field trials after five days. In addition, *P. tundrae* M4 ranked second, achieving 90.49–84.26% mortality in the lab and 87.87–83.81% in the field after one week. *S. tricolor* HM10 produced 80.06–74.09% mortality in laboratory assays and 76.73–73.36% under the field conditions. Effects on the predatory mites *Agistemus exsertus* and *Amblyseius swirskii* were minimal, with mortality ranging from 13.28 to 18.55% in the lab work and 12.46–16.74% in the field experiment. Genome analysis of strain HM10 revealed a biosynthetic gene cluster with predicted terpenes production. Terpenes can cause chemo-osmotic stress and broad membrane-disrupting capabilities. These results highlight the promise of microbial agents for sustainable mite management and provide a foundation for further optimization of bacterial biocontrol strategies.

## 1. Introduction

The olive tree (*Olea europaea* L.) is a subtropical evergreen tree that grows in temperate climates and can be cultivated in arid areas. It is native to the Mediterranean Basin, known for its silvery-green leaves and gnarled trunk. In Saudi Arabia, more than 21.5 million olive trees produce 351,000 tonnes annually. This significant number is a result of the application of sustainable agricultural practices and the expansion of cultivated areas [1]. However, olive trees are prone to being attacked by several phytophagous mites, including the olive bud mite *Oxycenus niloticus* (Zaher and Abou-Awad), the olive rust mite *Tegolophus hassani* Keifer, the olive mite *Aceria olivi* Zaher and Abou-Awad (Acari: Eriophyidae), and the spider mite, *Tetranychus urticae* Koch (Acari: Tetranychidae), resulting in serious damage and significant reductions in yield [2,3]. The heavy use of chemical pesticides, including carbamates and organophosphates, has led to significant economic losses and risks to environmental and human health and adversely affected natural enemies, the presence of pesticide residues in crops, increasing resistance among pests, making them less effective over time, causing soil degradation, abatement of biodiversity as well as the negative impacts on life health. This has raised concerns about the sustainability of chemical control methods in agriculture [4,5,6,7]. These concerns mainly require the development of alternative pest control techniques, such as microbial control agents for the management of mites infesting olive trees. The creation of biopesticides is an alternative strategy to reduce reliance on these dangerous chemicals [8,9]. The use of biological pesticides provides the best substitute that is easy to adopt, cost-effective, safe, and successful against different pathogens and pests [10]. The Environmental Protection Agency (EPA) of the United States of America states that microbial biopesticides are pest pathogenic microbiomes that were substances derived from organisms and possess the capacity to eliminate pests through infection [11]. Biopesticides attained from the organisms (fungi, bacteria, nematodes, protozoan, and viruses) can be broadly utilized to control the population of mites, insects, plant pathogens, nematodes, and weeds [12,13,14,15,16]. Approximately 74% of biopesticides produced globally come from bacterial microbiomes [17]. Numerous entomopathogenic bacteria species have the capacity to cause infection as well as harm to mites and insects [12,18]. Applications of entomopathogenic bacteria have been utilized under the IPM program, which is frequently applied for the management of diseases and pests [19]. Numerous insect and mite killing compounds, namely avermectin, abamectin, emamectin, spinosyns, polynactins, and milbemycin, were isolated from Actinomycetes and have the potential to cope with mite and insect pest problems in agriculture [20].

Numerous studies have confirmed that biopesticides are selective and safe for non-targeted organisms as well as for the environment [21]. In addition, biopesticides are cheap and economical, easy to produce, eco-friendly, very effective, and could control the pest population and show various strategies to cope with pest resistance [22].

In Saudi Arabia, several investigations were conducted on the biological control of the citrus rust mite *Phyllocoptruta oleivora* (Ashmead) by three bacterial preparations of *Streptomyces tricolor* strain HM10, *Streptomyces thinghirensis* strain HM3, and *Bacillus subtilis* E5 under field and laboratory conditions [12]. In addition, other studies were carried out on the biological control of the spider mite, *T. urticae*, by three bacterial preparations of *Acinetobacter* sp., *B. qassimus*, and *Bacillus subtilis* [23]. Nevertheless, no study to date has examined the possibility of *Bacillus subtilis* D3, *Paenibacillus tundrae* strain M4, and *Streptomyces tricolor* strain HM10 on phytophagous mites infesting olive trees. *B. subtilis* D3 is a soil-dwelling, Gram-positive, rod-shaped bacterium, producing endospores with dimensions of 2–6 µm × <1 µm. Its robust peptidoglycan cell wall and spore structures underpin its survival in diverse and harsh environments; it is known for its beneficial effects on plant health and its role as a biological nematicide in soil, suppressing numerous plant-parasitic nematodes, including cyst nematodes and root-knot nematodes [24,25,26]. *Paenibacillus* species have been known as a pathogen of nematodes, mollusks, and lepidopteran insects [27,28].

*Paenibacillus tundrae* is categorized as a Gram-positive, spore-forming bacterium, rod-shaped, encircled with flagella, and can develop and grow in both aerobic and anaerobic environments. Its morphology supports resilience in cold, acidic, and variable-oxygen environments. *Streptomyces tricolor* is a Gram-positive, filamentous Actinobacterium characterized by branching substrate and aerial hyphae, spore-chain formation, and pigmented, mycelium-rich colonies. Its morphology aligns with the classical *Streptomyces* developmental cycle, including hyphal differentiation and sporulation, and reflects its adaptation to soil environments. The bacterium *S. tricolor* is well-known for its biological control activity and ability to generate a variety of secondary metabolites, including siderophores, which are crucial for promoting plant growth [29]. The interest in employing plant-growth-promoting bacteria (PGPB) as biological control agents is significantly growing due to the discovery of new properties of certain beneficial bacteria in safeguarding crops, resulting in a significant decrease in the use of chemical pesticides. Therefore, the purpose of the current study was to evaluate the impact of the following three bacterial species: *B. subtilis*, *P. tundrae*, and *S. tricolor* against mites infesting olive trees, and their impact on predatory mites *A. exsertus* and *A. swirskii* under laboratory and field conditions.

## 2. Results

### 2.1. Effect of B. subtilis, P. tundrae, and S. tricolor on the Mobile Stages and Eggs of O. niloticus, T. hassani, A. olive and T. urticae

The three bacterial species *B. subtilis* D3, *P. tundrae* M4 and *S. tricolor* HM10 were tested against the eggs and mobile stages of *Oxycenus niloticus*, *Tegolophus hassani*, *Aceria olivi* and *Tetranychus urticae* under field and laboratory conditions, besides their detrimental effects on the predatory mites, *Agistemus exsertus* and *Amblyseius swirskii*.

The highest effectiveness rate on *O. niloticus*, *T. hassani*, *A. olivi* and *T. urticae* was observed with *B. subtilis* sprays. Five days after treatment, mite mortality was 91.84, 90.27, 89.75 and 85.36 under lab conditions and 88.90, 89.51, 88.24, and 84.12% under field conditions, followed by *P. tundrae*, which demonstrated miticidal action against *O. niloticus*, *T. hassani*, *A. olivi* and *T. urticae*. One week after treatment, mite mortality reached 90.49, 89.24, 86.37, and 84.26 under lab conditions and 87.87, 88.17, 85.10, and 83.81% under field conditions. Moreover, the mortality rates were noticeably lower, 80.06, 78.10, 75.60, and 74.09; 76.73, 77.50, 74.23, and 73.36% using *S. tricolor* under lab and field conditions, seven days after treatment (Table 1, Table 2, Table 3 and Table 4).

The findings of acaricidal activity of the three bacterial species suggest that the application of *B. subtilis* D3, *P. tundrae* strain M4, and *S. tricolor* strain HM10 be regarded as a potentially effective control agent against the mobile stages of *O. niloticus*, *T. hassani*, *A. olivi*, and *T. urticae*. The current investigation documented the following diagnostic characteristics, which are thought to be general features of bacterial infection: incapacity to move, food discontinuation, low rate of excretion following feeding stop, deterioration of the external cuticle, discharge of diarrhea-like feces, noticeable color variations (dark gray tinged with black), and dead mites become light black and soft as a result of the bacterial infection. In the case of *P. tundrae* strain M4, the infected mites become milky as the bacterium expands all over the body. By the end of the infection phase the milky color turned to light gray.

### 2.2. The Effects of Three Bacterial Preparations on the Predacious Mites, Agistemus exsertus and Amblyseius swirskii

Despite the eriophyid and spider mite being adversely impacted by being exposed to the three bacterial species, the predatory mites, *A. exsertus* and *A. swirskii*, were less impacted. The number of mobile stages was calculated daily for one week to observe the cumulative decrease in *A. exsertus* and *A. swirskii*. Mortality rates continue to rise for the next five days for *B. subtilis* D3, *P. tundrae* strain M4; whereas, for the *S. tricolor* strain HM10 treatment, the fatality rate continued to rise until day seven. A statistical analysis revealed that the mortality rates of *A. exsertus* and *A. swirskii* populations showed no discernible differences across the three treatments (Table 5 and Table 6). The results showed that the predacious mites, *A. exsertus* and *A. swirskii*, showed a greater ability for tolerance than phytophagous mites to the three bacterial species (*p* < 0.001). The corrected mortality percentages were 15.35, 14.70, and 13.28%; 18.55, 16.85, and 15.33% under lab conditions and 14.75, 13.29, and 12.46%; 16.74, 15.42, and 14.26% under field conditions after one week of exposure with *B. subtilis*, *P. tundrae*, and *S. tricolor*, respectively.

The following are the pathological symptoms of the predatory mite that were observed throughout this investigation: before they died, individuals of *A. exsertus* and *A. swirskii* had a characteristic dark-gray gut blockage and became so pale that they were translucent.

### 2.3. Assessment of the Three Bacterial Species on O. niloticus, T. hassani, A. olivi, and T. urticae Egg Hatchability

The results of this study showed that the hatchability of the eggs in the untreated control increased exponentially, ranging from 96.95 to 98.28%, by day 5 of the observation period, which was higher than the hatch rates stated in the treatment groups, as shown in Table 7.

Conversely, *B. subtilis* D3, *P. tundrae* strain M4 and *S. tricolor* strain HM10 demonstrated a successful inhibition of egg hatchability, which was reduced to 27.56, 36.31, and 44.21%; 30.54, 35.76 and 45.87%; 31.17, 32.88 and 42.46 and 35.32, 37.41 and 46.48 of laid eggs by *O. niloticus*, *T. hassani*, *A. olive* and *T. urticae*, respectively, after the period of five days which is significantly lower as compared to control (98.28, 96.95, 97.84 and 98.14), indicating inhibition of hatching after being exposed to these strains. The rates of egg hatchability in the control group and the groups treated with *B. subtilis*, *P. tundrae*, and *S. tricolor* strains showed significant differences. This distinction highlights the pronounced acaricidal efficacy of three bacterial strains, thus underscoring their potential as a useful tool in reducing *O. niloticus*, *T. hassani*, *A. olivi*, and *T. urticae* egg hatchability. The following are the pathological symptoms of the eggs that were observed throughout this investigation: before they failed to hatch, eggs of *O. niloticus*, *T. hassani*, *A. olivi* had a shrinking of the eggshell with the egg turning a light gray color, which eventually turns black over time. Meanwhile, in the case of *T. urticae* eggs, the color of the eggs gradually changed from transparent to light yellow and then to gray.

### 2.4. Phylogenetic Tree and Genome Analysis

**Figure 1:**
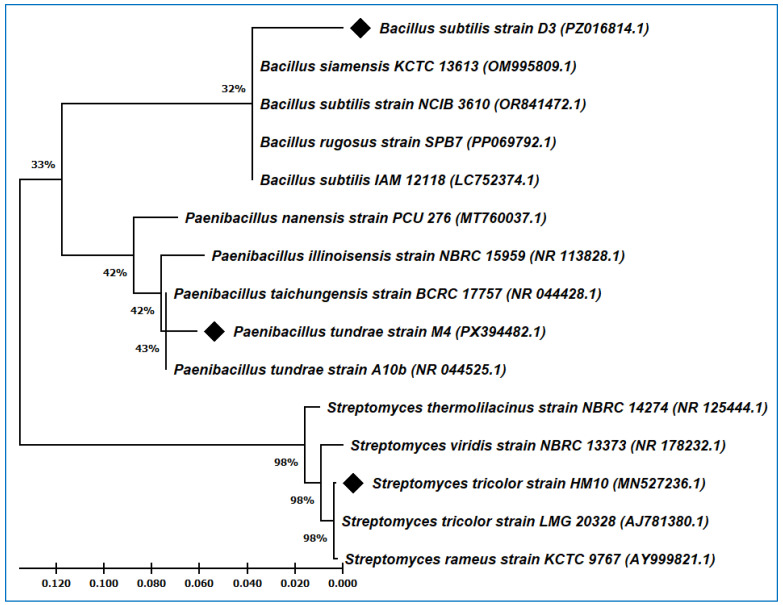
The molecular phylogenetic tree of *Paenibacillus tundrae* M4, *Bacillus subtilis* D3 and *Streptomyces tricolor* HM10 with their closely related strains in the GenBank based on the 16S rRNA sequences. (♦) symbol pointed to the strains under study.

The phylogenetic tree was constructed using the 16S rRNA gene sequences from the identified strains, with their closely related strains in the GenBank. The BLAST (version: 2.17.0) search of strain *Paenibacillus tundrae* M4 displayed 97.68% identity with the type strain *Paenibacillus tundrae* A10b, whereas *Bacillus subtilis* strain D3 revealed 96.96% similarity with the type strain *Bacillus subtilis* 168. In addition, strain HM10 exhibited a close relationship with the type strain *Streptomyces tricolor* LMG 20328 and showed the highest degree of similarity, reaching 99.69%. When the 16S rRNA sequences from the three applied strains and their related sequences from the GenBank were aligned and used to construct the phylogenetic tree, *P. tundrae* M4 branched off separately with their related *Paenibacillus* strains in one group (Figure 1). Furthermore, *B. subtilis* strain D3 followed the same direction and grouped with other *Bacillus* strains, whereas *S. tricolor* HM10 clustered with its closer strains from the genus *Streptomyces*.

**Figure 2:**
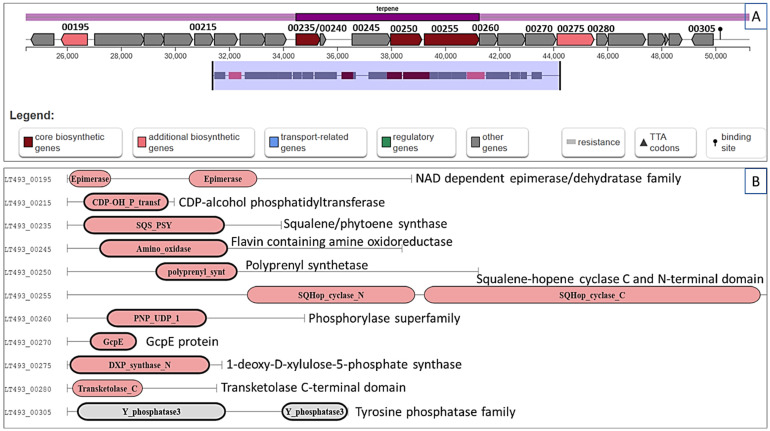
Terpene (hopene) biosynthetic gene cluster in *S. tricolor* HM10. (**A**) The proposed terpene gene cluster in region 2.1 from HM10 genome; (**B**) the predicted genes involved in terpene biosynthesis based on Pfam domain annotation. The main genes in terpene biosynthesis are: squalene synthase HpnC (LT493_00235), squalene synthase HpnD (LT493_00240), hydroxysqualene dehydroxylase HpnE (LT493_00245), polyprenyl synthetase (LT493_00250), squalene hopene cyclase (LT493_00255), 1-hydroxy-2-methyl-2-butenyl 4-diphosphate reductase (LT493_00260), and 1-deoxy-D-xylulose-5-phosphate synthase (LT493_00275).

To predict the potential secondary metabolites related to the mite species control, the whole genome of *S. tricolor* HM10 was subjected to antiSMASH analysis. A potential biosynthetic gene cluster related to terpene biosynthesis was identified inside *S. tricolor* HM10 genome (Figure 2). This cluster locates in region 2.1 among nucleotides 24,474 and 51,258 (total: 26,785 nt in size). The proposed gene cluster presented a similarity score of 0.55 to the terpene (hopene) biosynthetic gene cluster from *Streptomyces coelicolor* A3(2). This biosynthetic gene cluster (BGC) segment had up to 24 genes and most of them are involved in terpene biosynthesis (Figure 2A). The main putative genes in the biosynthesis process are UDP-glucose 4-epimerase GalE (LT493_00195), which is predicted to catalyze the reversible interconversion of UDP-glucose and UDP-galactose. In addition, squalene synthase HpnD (LT493_00240) is supposed to catalyze the formation of presqualene diphosphate (PSPP) from two molecules of farnesyl diphosphate (FPP), whereas squalene synthase HpnC (LT493_00235) will function in converting presqualene diphosphate (PSPP) to hydroxysqualene (HSQ). Hydroxysqualene dehydroxylase HpnE (LT493_00245) is predicted to reduce hydroxysqualene (HSQ) to squalene (SQ). On the other hand, polyprenyl synthetase family protein (LT493_00250) is proposed to elongate the linear hydrocarbon backbones (prenyl diphosphates) from five-carbon isoprene units (isopentenyl diphosphate and dimethylallyl diphosphate), whereas squalene hopene cyclase (LT493_00255) works in inverting the linear precursor squalene into pentacyclic triterpenes through the cyclization process. Furthermore, 1-hydroxy-2-methyl-2-butenyl 4-diphosphate reductase (LT493_00260) will convert 1-hydroxy-2-methyl-2-(E)-butenyl 4-diphosphate into DMAPP (dimethylallyl diphosphate) and IPP (isopentenyl diphosphate). Additionally, 1-deoxy-D-xylulose-5-phosphate synthase (LT493_00275) and transketolase C-terminal domain (LT493_00280) are supposed to be involved in the biosynthesis of 1-deoxy-D-xylulose 5-phosphate (DXP) from pyruvate.

## 3. Discussion

Microorganisms produce useful natural products, including bioactive substances and natural antibiotics that have a variety of uses. These microorganisms, such as pathogenic bacteria, provide a much safer and environmentally friendly alternative to the commercially available chemical pesticides [30]. *Streptomyces* species, which are actinobacteria, have produced a wide range of secondary metabolites with significant commercial value. The detection of the avermectins from *Streptomyces avermitilis* as strong acaricides and antiparasitic agents accelerated the search for more novel pesticidal [31].

In continuation of that, the acaricidal potential of *Streptomyces tricolor* strain HM10, *Bacillus subtilis* D3, and *Paenibacillus tundrae* strain M4 was assessed in the current study against *O. niloticus*, *T. hassani*, *A. olivi*, and *T. urticae* under lab and field conditions. In comparison to control, significantly higher mobile stage mortality and lower egg hatching were noted after treatment with these three strains. In 2025, Al-Azzazy et al. [12] stated that *S. tricolor* HM10 reduced egg hatching in the citrus rust mite *Phyllocoptruta oleivora* (Ashmead) to 36.74% and induced mobile stages mortality to 83.66% after six days. In another report, Kaur et al. [32] displayed that culture filtrates of *Streptomyces hydrogenans* strain DH16 were found to be more lethal than to *Meloidogyne incognita* because 90% of J2 mortality occurred, and only less than 1% egg hatching in the time period of 72 h. Similar results were obtained by Al-Azzazy et al. [23], who reported that the direct application of *Bacillus qassimus*, *Bacillus subtilis*, and *Acinetobacter* sp. on two spotted spider mite, *Tetranychus urticae*, caused a mite mortality of 70.74%, 72.22%, and 84.11%, respectively, under laboratory conditions.

Pathogenic bacteria invade their hosts primarily through the alimentary tract and mouth parts. After that, tripartite Tc toxin complexes of pathogenic bacteria pierce the midguts of the host and introduce toxic enzymes as soluble proteins inside the host cell. Due to the midgut’s high pH, the poisons dissolve and are broken down by gut proteolytic enzymes into their toxic moieties [33,34]. In other instances, pathogenic bacteria enter via the integument, which damages the exoskeleton’s essential function in the hosts, leading to fatality [35,36]. *Bacillus subtilis* D3 is demonstrated to possess strong nematocidal properties; this is therefore of interest to scientists nowadays. Jurat-Fuentes and Jackson [37] reported that the soil bacterium *B. firmus* was described as pathogenic against larvae of the narcissus moth, *Eligma narcissus* Cramer (Lepidoptera: Nolidae). Dead larvae become black and soft, and discharged gut contents contain the rod-shaped bacterium and symptomatic of septicemia. In addition, *Bacillus* species exhibits a certain amount of toxicity by the production of endotoxins during sporulation; the receptors on intestinal epithelial cells in the midgut react with crystalliferous inclusions to release δ-endotoxins. Then, the pest’s midgut is harmed by the proteinaceous toxins produced by bacteria during the late infectious stage, with the ensuing osmotic imbalance leading to rupture of cells, jeopardizing the gut epithelium’s integrity, resulting in the death of the mite and different insect pests [23,38,39].

In the present study, numerous elements may have played a part in the mortality of *O. niloticus*, *T. hassani*, *A. olivi*, and *T. urticae*. In another study, Wilson et al. declared that external toxins called bacterial hemolysins target the membranes of cells, resulting in bodily distension and cell tears. These hemolysins might also be a factor in the pathogenicity of *S. tricolor* strain HM10, *B. subtilis* D3 and *P. tundrae* strain M4 against *O. niloticus*, *T. hassani*, *A. olivi*, and *T. urticae*, especially given the rapid death rate monitored.

*Paenibacillus tundrae* belongs to the genus *Paenibacillus*, which was divided from *Bacillus* in 1993 due to its unique genetic and behavioral traits. Members of this genus have been known as an obligatory pathogen of nematodes, insects, mollusks, phytopathogenic bacteria, and fungi [40,41]. *Paenibacillus popilliae* was discovered to infect Japanese beetle Popillia japonica Newman and was registered as the first microbial control agent in US [42]. According to Grady et al. [43], these bacteria create the enzyme chitinase, which hydrolyzes chitin, a structural polysaccharide of insect exoskeleton and stomodeum and proctodeum, as well as other structures., resulting in insect death. Numerous studies have emphasized the enormous application potential of *Paenibacillus* in several fields, including plant growth promotion, nitrogen fixation, hormone generation, phosphate solubilization, bioremediation of xenobiotics, and biocontrol activities [44].

Compared to other ecosystem interventions, the threat to natural enemies should be evaluated while assessing the pathogenic bacteria’s safety and environmental impact [45]. The three bacterial species showed preferential toxicity towards the phytophagous mites while saving the predatory mites. The obtained mortality values for predacious mites are significantly different and were five to six times higher than the corresponding ones for phytophagous mites. This could be attributed to the fact that predatory mites possess a thicker body wall with shields on the ventral and dorsal sides [46] that may obstruct the penetration of the bacteria into their bodies and decrease the received dose of bacteria compared to the thin-walled body of plant-feeding mites [12].

Our study revealed that the three bacterial species tested inhibit egg hatching under laboratory conditions. These impacts align with other findings, against citrus rust mite *P. oleivora*, including those of the *Streptomyces tricolor* strain HM10 that totally inhibits egg hatching to 36.74% [12]. In 2019, d’Errico et al. [24] stated that *B. firmus* has shown a lethal effect on nematodes such as *Meloidogyne incognita*, *Tylenchulus semipenetrans*, *Heterodera* sp., *Ditylenchus* sp., *Xiphinema index*, *Pseudopyrenochaeta lycopersici* and *Rodopholus similis*, where the toxins created by *B. subtilis* cause damage to the gall-forming nematodes’ outer egg pellicle, thus avoiding the hatching of plant parasitic nematode eggs. Furthermore, *S. hydrogenans* strain DH16 was found to be more lethal to *M. incognita* because of only less than 1% egg hatching in the period of three days [32].

When going forward and analyzing the *S. tricolor* MH10 genome, a predicted pathway was identified related to terpenes biosynthesis. Terpenes are natural products that have a wide range of structural variations, such as carbocyclic skeletons or linear hydrocarbons. Terpenes are widespread in the genus *Streptomyces* and have antiviral, antifungal, antibacterial, and antiparasitic properties [47]. They are employed as insecticides to store agricultural products [48,49]. Li et al. [50] evaluated the effectiveness of six terpenes that are frequently present in essential oils (carvacrol, eugenol, geraniol, citral, terpinen-4-ol, and linalool) against *Sarcoptes scabiei* eggs. They found that significant ovicidal actions are exhibited by carvacrol, eugenol, and geraniol, which make them promising ovicidal drugs for scabies treatment. In addition, combinations from three terpenes (carvacrol, thymol, and menthol) were assessed on the poultry red mite (*Dermanyssus gallinae*) under in vitro and in-field assays.

This terpene-based combination demonstrated encouraging miticidal action under in vitro and field application [51,52]. Wei et al. [53] used the highly pure chemical xenocoumacin 1 (purified from the supernatant of the *Xenorhabdus nematophila* CB6) to investigate its acaricidal effects on *T. urticae* and its predator *Neoseiulus californicus*. According to a number of research, monoterpenes mainly target insects’ nervous systems to produce their insecticidal effects [54]. Thymol (monoterpenoid) exhibited acaricidal activity through reducing wing-beat frequency and flight muscle impulses by interfering with the energy metabolism, electrical activity of the dorsal longitudinal flight muscles and nerve conduction of the mites [55,56,57]. Eugenol has neuroinhibitory effects, whereas linalool possesses neuroexcitatory characteristics [58]. Within the insect nervous system, monoterpenes may target ion channels like γ-aminobutyric acid type A receptors (GABAARs), nicotinic acetylcholine receptors (nAChRs), tyramine (TA) receptors, octopamine (OA) receptors, transient receptor potential (TRP) channels, in addition to enzymes such as acetylcholinesterase (AChE) and Na+/K+-ATPase [59,60].

## 4. Materials and Methods

### 4.1. Bacterial Strains and Culture Conditions

The previously isolated and identified *Streptomyces tricolor* strain HM10 (Accession, MN527236) [61] was used in this study, in addition to new isolates (*B. subtilis* D3 and *P. tundrae* M4 isolated from the soil), which were grown in glucose soyabean meal broth (GSB) and Nutrient Broth (NB) media, respectively. The three bacterial strains were inoculated on the appropriate medium and incubated at 30 °C with shaking at 150 rpm for 3 days. The bacterial cells (approximately with OD_600_ = 1.12) were removed from the culture filtrates through the Whatman Filter Paper (Grade 1), and the produced filtrates were implemented in either the lab or field against four mite species, *O. niloticus*, *T. hassani*, *A. olivi*, and *T. urticae*.

### 4.2. Bacterial Identification and Phylogenetic Tree

The bacterial genomic DNA from D3 and M4 isolates was extracted according to the method of Cook and Meyers [62]. DreamTaq PCR Master Mix (Thermo Scientific, Vilnius, Lithuania) was used to amplify the 16S rRNA region using general bacterial identification primers (27F and 1492R). The resulting PCR product was purified, sequenced (Macrogen, Seoul, Republic of Korea), assembled (BioEdit V7.7.1), and deposited in the GenBank under the following accession numbers: PZ016814 and PX394482. Using ClustalW built in MEGA X software (version: 10.2.6) [63], the 16S rRNA sequences of *Bacillus subtilis* strain D3, *Paenibacillus tundrae* strain M4, and *Streptomyces tricolor* strain HM10 were blasted and aligned with closely related species in the GenBank. The Tamura–Nei model, Maximum Likelihood, and Bootstrap method deduced the phylogenetic tree using the sequences obtained from the BLAST search in the NCBI database.

### 4.3. Genome Analysis and Potential Gene Cluster Identification

The available genome sequence of *S. tricolor* HM10 (GenBank: JA-JREA000000000.1) was subjected to analysis by antiSMASH (bacterial version, antiSMASH 7.0, accessible on 9 March 2025) [64]. In addition, unknown secondary metabolite biosynthesis gene clusters were identified using PRISM (Prediction Informatics for Secondary Metabolomes), in addition to their projected chemical structures [65]. Furthermore, the MIBiG (Minimum Information about a Biosynthetic Gene cluster) was applied for cluster comparison with identified known gene clusters in NCBI GenBank [66].

### 4.4. Lab Experiments—Residual Effects on Moving Stages and Eggs of O. niloticus, T. hassani, A. olivi, and T. urticae

All experiments were conducted at the laboratory of entomology, Department of Plant Protection, College of Agriculture and Food, Qassim University, Saudi Arabia. Naturally infested olive leaves were obtained from the agricultural research and experimental station of Qassim University, Saudi Arabia. Leaves were infected with *O. niloticus*, *T. hassani*, *A. olivi*, and *T. urticae*. Pre-treatment counts all laid eggs, and the mite’s mobile individuals were noted before treatment. Olive leaves were directly sprayed with 100 milliliters of the bacterial preparations of *Bacillus subtilis* D3, *Paenibacillus tundrae* strain M4, and *Streptomyces tricolor* strain HM10 using a high-pressure hand pump sprayer. Only tap water was sprayed on the control group. Olive leaves were placed upside down; the upper surfaces were placed face down on cotton wool that had been moistened (0.9 cm thick) in Petri dishes (16 cm in diameter × 2.5 cm high), one leaf for each Petri dish. A ring of Vaseline was applied to the leaf margins to prevent mites from escaping. Every day, 10 milliliters of water were given to the Petri dish to keep the cotton moist. Translucent plastic boxes (L 30 × W 25 × H 20 cm) were used to hold the Petri dishes and glass and were placed in an incubator at 30 ± 1 °C, 55 ± 5% RH, and a photoperiod of 14 h light (14 L)/10 h dark (10D). For each bacterial species, three replicates were performed. Olive leaves were checked daily, and the number of dead mites was recorded during seven days. The hatching of laid eggs was documented every day, where hatched protonymphs of experimental species were counted daily until the end of hatching in the untreated control, and hatched protonymphs were removed daily.

### 4.5. Laboratory Experiments—Direct Impacts on Predatory Mites, Agistemus Exsertus and Amblyseius swirskii

Colonies of predatory mites, *A. exsertus* and *A. swirskii*, were established with individuals collected from unsprayed olive orchards in Al Mulayda, Al-Qassim region (26.299138° N, 43.78896° E), Saudi Arabia. To obtain age-matched egg cohorts, eighty gravid females of each predator species extracted from the stock colonies were positioned in rearing units made of common bean leaves, *Phaseolus vulgaris* L. (Fabaceae), with about 800 two-spotted spider mites, *Tetranychus urticae*, as prey to each predator species. These mite-rearing units were maintained in an incubator at 32 ± 1 °C and 50 ± 5% RH. After two days, eggs laid by each predator were carefully moved separately to new rearing units, and the newly hatched larvae were provided with immature stages of spider mite, *T. urticae* as prey; suitable numbers of prey nymphs (20 *T. urticae* individuals) were added every day to the rearing units. Forty identically aged adult females of each predator species were carefully transferred to a bean leaf disk (4 cm in diameter), positioned face down in a Petri dish (8 cm in diameter × 2.5 cm high) on cotton that has been moistened with distilled water and sprayed with 10 mL of the bacterial preparations of *Bacillus subtilis* D3, *Paenibacillus tundrae* strain M4 and *Streptomyces tricolor* strain HM10 utilizing a tiny pressure spray bottle. For each bacterial species, three replicates were performed, and 30 adult females of each predator species were considered per replicate. Female mortality was noted 72 h after spraying. The control groups received merely tap water spraying. The bacterial incident was checked by stereo microscope inspection of *A. exsertus* and *A. swirskii* cadavers.

### 4.6. Field Experiment Impacts on Phytophagous and Predatory Mites

This study was conducted at the unsprayed olive orchards in Al Mulayda, Al-Qassim region (26.299138° N, 43.78896° E), Saudi Arabia. Four groups, each made up of three young olive trees (*Olea europaea* L.) (3 years old) of similar size and shape with a history of *O. niloticus*, *T. hassani*, *A. olivi*, and *T. urticae* infestations, were chosen for the study. Individual trees (replicates) were completely randomized as part of the experimental research design. For every bacterial species and untreated control, three replicates were employed. Three different species of bacteria, *B. subtilis* D3, *P. tundrae* strain M4 and *S. tricolor* strain HM10, were recently prepared from the stock culture (750 mL each with OD_600_ = 1.12) and immediately sprayed following filtration (750 mL from each strain culture) on olive trees (approximately 250 mL for each olive tree) using a one-liter volume hand atomizer for testing all mobile stages of *O. niloticus*, *T. hassani*, *A. olivi* and *T. urticae* and compared with the control, as well as their side impacts on the predacious mites, *A. exsertus* and *A. swirskii*. Tap water was sprayed onto the untreated control.

Twenty olive leaves per tree were randomly collected in all directions (60 leaves per treatment; 240 leaves per sampling). Leaves were placed in labeled plastic bags and transported to the acarology laboratory, where they were examined under a stereoscopic microscope. To ascertain the initial density and distribution of phytophagous and predacious mites, a pre-spray count was conducted for every replicate. Observations were conducted daily for seven consecutive days post-treatment.

### 4.7. Statistical Analysis

The proportion reduction in the mean populations of phytophagous and predacious mites and the mean proportion of the number of protonymphs (in the case of eriophyid mites) and larvae (in the case of *T. urticae*) hatching from eggs of *O. niloticus*, *T. hassani*, *A. olivi*, and *T. urticae* were determined using Henderson and Tilton’s equation 1955.

$$Corrected\;Mortality\%=(1-\frac{Cb\times Ta}{Ca\times Tb})\times 100$$

where *Cb* is the number of mites or laid eggs in the untreated control before spray, *Ca* is the number of hatched larvae and protonymphs or mites in the untreated control after spray, *Tb* is the number of mites or laid eggs in treatment before spray, and *Ta* is the number of hatched larvae and protonymphs or mites in treatment after spray.

The mortalities of *O. niloticus*, *T. hassani*, *A. olivi*, *T. urticae*, *A. swirskii*, and *A. exsertus* were determined by direct inspection. One-way analysis of variance (ANOVA) was used to statistically analyze the collected data for all variables. Tukey’s Honestly Significant Difference (HSD) test in Excel was applied to ascertain which specific group means are different from each other (*p* < 0.05).

## 5. Conclusions

The present study is the first investigation into acaricidal properties of *B. subtilis* D3, *P. tundrae* strain M4 against mites infesting olive trees. The current investigation revealed that *B. subtilis* D3, *P. tundrae* strain M4 are effective in managing *O. niloticus*, *T. hassani*, *A. olivi*, and *T. urticae* populations under laboratory and field conditions, while *Streptomyces tricolor* strain HM10 produces a moderate level of control. Consequently, pathogenic bacteria could be applied as a possible microbial control on *O. niloticus*, *T. hassani*, *A. olivi*, and *T. urticae* in the olive orchards, providing a practical substitute for traditional synthetic acaricides. Also, the three bacterial species reduced egg hatching around 53.52–64.68% in laboratory bioassays. Nevertheless, in addition to their mite-killing capabilities and inhibitory impacts on the eggs hatching, the three bacterial species exhibited a slight contact toxicity on the predatory mites, *A. exsertus* and *A. swirskii*, under laboratory and field conditions. Additionally, complementary studies should be carried out to assess the viability of these bacterial species in mite pest management plans as a means of satisfying the growing need for nonchemical control methods.

## Figures and Tables

**Table 1:** Corrected mortality (in % calculated with Henderson–Tilton equation) of the olive bud mite, *Oxycenus niloticus* treated with the following three bacterial species: *Bacillus subtilis* D3, *Paenibacillus tundrae* M4, *Streptomyces tricolor* HM10 under laboratory and field conditions.

Treatment	Under Laboratory Conditions at 30 ± 1 °C, 55 ± 5% RH							*p* Value
	Days Following Treatment							
	1	2	3	4	5	6	7	
*Bacillus subtilis* D3	34.19 Aa	47.60 Ab	58.11 Ac	73.49 Ad	91.84 Ae	90.61 Ae	90.20 Ae	0.0074
*Paenibacillus tundrae* M4	33.29 Aa	45.28 Ab	56.25 Ac	68.43 Ad	79.64 Be	89.15 Af	90.49 Af	0.0072
*Streptomyces tricolor* HM10	22.38 Ba	34.90 Bb	45.74 Bc	59.82 Bd	69.75 Ce	79.18 Bf	80.06 Bf	0.0066
*p* value	0.0053	0.0036	0.0042	0.0075	0.0050	0.0030	0.0025	
Under field conditions								
*Bacillus subtilis* D3	31.26 Aa	45.50 Ab	56.23 Ac	70.15 Ad	88.90 Ae	84.61 Ae	84.00 Ae	0.0055
*Paenibacillus tundrae* M4	30.44 Aa	44.37 Ab	53.30 Ac	65.51 Ad	77.29 Ae	86.27 Af	87.87 Af	0.0070
*Streptomyces tricolor* HM10	20.06 Ba	31.81 Bb	42.26 Bc	55.63 Bd	69.72 Be	72.34 Be	76.73 Be	0.0047
*p* value	0.0040	0.0067	0.0058	0.0071	0.0061	0.0048	0.0030	

The capital letter denotes significant differences within the same column, whereas the small letter denotes significant differences within the same row at *p* < 0.01.

**Table 2:** Corrected mortality (in % calculated with Henderson–Tilton equation) of the olive rust mite, *Tegolophus hassani* treated with the following three bacterial species: *B. subtilis* D3, *P. tundrae*, *S. tricolor* under laboratory and field conditions.

Treatment	Under Laboratory Conditions at 30 ± 1 °C, 55 ± 5% RH							*p* Value
	Days Following Treatment							
	1	2	3	4	5	6	7	
*Bacillus subtilis* D3	29.09 Aa	41.16 Ab	56.61 Ac	69.53 Ad	90.27 Ae	88.99 Ae	86.95 Ae	0.0094
*Paenibacillus tundrae* M4	26.58 Aa	39.51 Ab	53.50 Ac	64.76 Ad	75.46 Be	86.77 Af	89.24 Af	0.0086
*Streptomyces tricolor* HM10	16.11 Ba	26.44 Bb	37.75 Bc	47.00 Bd	59.12 Ce	70.00 Bf	78.10 Bf	0.0048
*p* value	0.0081	0.0038	0.0041	0.0092	0.0046	0.0087	0.0039	
Under field conditions								
*Bacillus subtilis* D3	25.16 Aa	36.77 Ab	58.18 Ac	76.62 Ad	89.51 Ae	88.10 Ae	88.00 Ae	0.0079
*Paenibacillus tundrae* M4	23.35 Aa	36.38 Ab	47.45 Ac	58.00 Bd	70.56 Be	84.33 Af	88.17 Af	0.0058
*Streptomyces tricolor* HM10	12.49 Ba	25.32 Bb	36.77 Bc	49.84 Cd	60.59 Ce	71.82 Bf	77.50 Bf	0.0067
*p* value	0.0061	0.0088	0.0049	0.0073	0.0092	0.0065	0.0084	

The capital letter denotes significant differences within the same column, whereas the small letter denotes significant differences within the same row at *p* < 0.01.

**Table 3:** Corrected mortality (in % calculated with Henderson–Tilton equation) of the olive mite, *Aceria olivi* treated with the following three bacterial species: *B. subtilis* D3, *P. tundrae*, *S. tricolor* under laboratory and field conditions.

Treatment	Under Laboratory Conditions at 30 ± 1 °C, 55 ± 5% RH							*p* Value
	Days Following Treatment							
	1	2	3	4	5	6	7	
*Bacillus subtilis* D3	27.30 Aa	38.42 Ab	52.17 Ac	69.53 A	89.75 Ad	88.15 Ad	87.36 Ad	0.0078
*Paenibacillus tundrae* M4	25.46 Aa	37.19 Ab	50.50 Ac	62.28 Ad	73.80 Be	84.29 Af	86.37 Af	0.0063
*Streptomyces tricolor* HM10	16.23 Ba	26.54 Bb	39.48 Bc	50.29 Bd	62.82 Ce	73.49 Bf	75.60 Bf	0.0070
*p* value	0.0094	0.0075	0.0062	0.0097	0.0050	0.0094	0.0061	
Under field conditions								
*Bacillus subtilis* D3	26.19 Aa	37.29 Ab	49.02 Ac	71.68 Ad	88.24 Ae	88.00 Af	86.16 Af	0.0085
*Paenibacillus tundrae* M4	22.37 Aa	36.49 Ab	48.70 Ac	59.27 Bd	70.14 Be	83.46 Af	85.10 Af	0.0074
*Streptomyces tricolor* HM10	15.36 Ba	25.16 Bb	37.44 Bc	48.40 Cd	59.68 Ce	72.96 Bf	74.23 Bf	0.0058
*p* value	0.0086	0.0071	0.0052	0.0078	0.0066	0.0047	0.0084	

The capital letter denotes significant differences within the same column, whereas the small letter denotes significant differences within the same row at *p* < 0.01.

**Table 4:** Corrected mortality (in % calculated with Henderson–Tilton equation) of the spider mite, *Tetranychus urticae* treated with the following three bacterial species: *B. subtilis* D3, *P. tundrae*, *S. tricolor* under laboratory and field conditions.

Treatment	Under Laboratory Conditions at 30 ± 1 °C, 55 ± 5% RH							*p* Value
	Days Following Treatment							
	1	2	3	4	5	6	7	
*Bacillus subtilis* D3	14.23 Aa	28.15 Ab	38.27 Ac	65.49 Ad	85.36 Ae	84.46 Ae	84.05 Ae	0.0058
*Paenibacillus tundrae* M4	12.39 Aa	27.14 Ab	36.91 Ac	49.53 Bd	58.28 Be	80.85 Af	84.26 Af	0.0047
*Streptomyces tricolor* HM10	5.45 Ba	15.27 Bb	25.48 Bc	36.88 Cd	47.19 Ce	68.14 Bf	74.09 Bf	0.0070
*p* value	0.0084	0.0068	0.0079	0.0062	0.0055	0.0075	0.0085	
Under field conditions								
*Bacillus subtilis* D3	14.00 Aa	30.08 Ab	49.18 Ac	61.29 Ad	84.12 Ae	83.00 Ae	82.87 Ae	0.0054
*Paenibacillus tundrae* M4	11.46 Aa	27.36 Ab	45.22 Ac	59.38 Ad	79.67 Ae	82.45 Ae	83.81 Ae	0.0061
*Streptomyces tricolor* HM10	9.29 Ba	14.49 Ba	24.34 Bb	37.67 Bc	51.18 Bd	62.85 Be	73.36 Be	0.0061
*p* value	0.0083	0.0090	0.0035	0.0047	0.0043	0.0074	0.0060	

The capital letter denotes significant differences within the same column, whereas the small letter denotes significant differences within the same row at *p* < 0.01.

**Table 5:** Corrected mortality (in % calculated with Henderson–Tilton equation) of the predacious mite, *Agistemus exsertus* treated with the following three bacterial species: *B. subtilis* D3, *P. tundrae*, *S. tricolor* under laboratory and field conditions.

Treatment	Under Laboratory Conditions at 30 ± 1 °C, 55 ± 5% RH						
	Days Following Treatment						
	1	2	3	4	5	6	7
*Bacillus subtilis* D3	0.00 Aa	2.00 Aa	7.73 Ab	12.29 Ac	15.70 Ad	15.24 Ad	15.35 Ad
*Paenibacillus tundrae* M4	0.00 Aa	2.05 Aa	7.12 Ab	11.46 Ac	14.90 Ad	14.55 Ad	14.72 Ad
*Streptomyces tricolor* HM10	0.00 Aa	0.00 Aa	2.07 Ba	7.21 Bb	10.62 Bc	13.11 Ac	13.28 Ac
Under field conditions							
*Bacillus subtilis* D3	0.00 Aa	3.10 Ab	6.14 Ac	10.20 Ad	14.95 Ae	14.63 Ae	14.54 Ae
*Paenibacillus tundrae* M4	0.00 Aa	2.25 Aa	5.00 Ab	8.80 Ac	13.80 Ad	13.25 Ad	13.20 Ad
*Streptomyces tricolor* HM10	0.00 Aa	0.00 Aa	2.15 Ba	5.23 Bb	9.87 Bc	12.14 Ac	12.46 Ac

The capital letter denotes significant differences within the same column, whereas the small letter denotes significant differences within the same row at *p* < 0.01.

**Table 6:** Corrected mortality (in % calculated with Henderson–Tilton equation) of the predacious mite, *Amblyseius swirskii* treated with the following three bacterial species: *B. subtilis* D3, *P. tundrae*, *S. tricolor* under laboratory and field conditions.

Treatment	Under Laboratory Conditions at 30 ± 1 °C, 55 ± 5% RH						
	Days Following Treatment						
	1	2	3	4	5	6	7
*Bacillus subtilis* D3	0.00 Aa	0.00 Aa	5.34 Ab	9.29 Ac	18.55 Ad	18.62 Ad	18.45 Ad
*Paenibacillus tundrae* M4	0.00 Aa	0.00 Aa	4.00 Ab	9.25 Ac	16.85 Ad	16.28 Ad	16.05 Ad
*Streptomyces tricolor* HM10	0.00 Aa	0.00 Aa	2.12 Ba	5.09 Bb	10.98 Bc	14.11 Ad	15.33 Ad
Under field conditions							
*Bacillus subtilis* D3	0.00 Aa	0.00 Aa	5.12 Ab	9.15 Ac	16.74 Ad	16.36 Ad	16.11 Ad
*Paenibacillus tundrae* M4	0.00 Aa	0.00 Aa	4.92 Ab	8.50 Ac	15.42 Ad	15.23 Ad	15.19 Ad
*Streptomyces tricolor* HM10	0.00 Aa	0.00 Aa	2.00 Ba	4.19 Ba	9.05 Bb	13.96 Ac	14.26 Ac

The capital letter denotes significant differences within the same column, whereas the small letter denotes significant differences within the same row at *p* < 0.01.

**Table 7:** Number of protonymphs and larvae hatching from eggs of *O. niloticus*, *T. hassani*, *A. olivi*, and *T. urticae*, treated with three bacterial species: *B. subtilis* D3, *P. tundrae*, *S. tricolor* under laboratory conditions.

Treatment	No. of Eggs, Protonymphs and Larvae/Leaf											
	No. of Eggs Pre-Spray Count				Average Number of Protonymphs and Larvae Post-Spray Count *				Hatching (%) **			
	*O. niloticus*	*T. hassani*	*A. olivi*	*T. urticae*	*O. niloticus*	*T. hassani*	*A. olivi*	*T. urticae*	*O. niloticus*	*T. hassani*	*A. olivi*	*T. urticae*
Control	74.68	81.07	90.10	68.37	73.40	78.60	88.16	67.10	98.28 a	96.95 a	97.84 a	98.14 a
*Bacillus subtilis* D3	76.45	88.16	85.24	70.19	20.71	26.10	26.00	24.33	27.56 b	30.54 b	31.17 b	35.32 b
*Paenibacillus tundrae* M4	80.61	76.28	79.63	65.37	28.77	26.45	25.62	24.00	36.31 b	35.76 b	32.88 b	37.41 b
*Streptomyces tricolor* HM10	70.07	80.45	94.81	72.61	30.45	35.78	39.46	33.12	44.21 c	45.87 c	42.46 c	46.48 c

* counts made one-week post treatment. ** hatching percentage calculated with Henderson–Tilton’s equation. Different letters in the vertical columns denote a significant difference (F-test, *p* < 0.01).

## Data Availability

Data are contained within the article.
